# The complete chloroplast genome sequences of three *Adenophora* species and comparative analysis with Campanuloid species (Campanulaceae)

**DOI:** 10.1371/journal.pone.0183652

**Published:** 2017-08-22

**Authors:** Kyeong-Sik Cheon, Kyung-Ah Kim, Ki-Oug Yoo

**Affiliations:** Department of Biological Sciences, Kangwon National University, Chuncheon, Gangwon, South Korea; Agriculture and Agri-Food Canada, CANADA

## Abstract

We report the complete chloroplast genomes of three *Adenophora* species, and analyzed these compared them to five published Campanuloid plastomes. The total genome length of *Adenophora divaricata*, *Adenophora erecta*, and *Adenophora stricta* ranged from 159,759 to 176,331 bp. Among the eight Campanuloid species, many inversions were found to be only in the LSC region. IR contraction was also identified in the plastid genome of *Adenophora stricta*. Phylogenetic analyses based on 76 protein coding genes showed that Campanuloids are monophyletic, and are composed of two major groups: *Campanula s*. *str*. and *Rapunculus*. When we compared each homologous locus among the four *Adenophora* species, ten regions showed high nucleotide divergence value (>0.03). Among these, nine loci, excepting *ycf3*-*rpoB*, are considered to be useful molecular markers for phylogenetic studies and will be helpful to resolve phylogenetic relationships of *Adenophora*.

## Introduction

Campanulaceae *s*. *str*. consists of approximately 1,046 species that are primarily distributed in temperate regions [1–4]. This family was divided into three groups, including Platycodonoids, Wahlenbergioids, and Campanuloids based on the capsule morphology [5], which was strongly supported by molecular phylogenetic studies based on nuclear ribosomal ITS (nrITS) and three chloroplast DNA (cpDNA) markers [4, 6–7].

The genus *Adenophora*, which belong to Campanuloids, is a perennial herbaceous plant with approximately 50–100 species that are distributed in temperate regions in Asia and Europe. This genus was first described by Fischer [8], and then, a classification system was established based on various studies [9–17] according to morphological characteristics, such as the phyllotaxy, presence or absence of petioles, shape of the calyx, and length of the disk. However, there are still many different opinions regarding the sections and subsections within the *Adenophora* classification system because very similar morphological characteristics are found the species. Additionally, many taxonomic studies [4, 6–7, 18–27] have been conducted in Campanulaceae, however, the phylogenetic relationships among the *Adenophora* remain to be elucidated.

Among the three *Adenophora* species discussed in this study, *A*. *erecta* is a very important species since it is an endemic species to Ulleung-do Island in Korea. This species was first described as a new species in 1997 [28] and is distinguished from *A*. *remotiflora* because the leaves are compactly arranged along the upper part of the stem, while the length of the disc is shorter than the width. Furthermore, *A*. *divaricata* and *A*. *stricta* are distributed in China, Japan, and Korea. In addition, *A*. *divaricata* differs from *Adenophora pereskiifolia* because the cylindrical disk is more than 1.2 times longer than the width, while the presence of trichome in the calyx tube is characteristic of *A*. *stricta* and distinguishe *A*. *stricta* from closely allied taxa, such as *Adenophora lamarckii* and *Adenophora polyantha* [29].

The structure of the chloroplast genome is highly conserved among land plants [30]. However, gene order changes due to rearrangements sometimes occur and can be used to obtain very important phylogenetic information [31–33]. Campanulaceae are known to have very different chloroplast genome structures in a genus or species due to many rearrangements [30–31, 34–38]. Therefore, the chloroplast genome structure of Campanulaceae is very useful for identifying their unclear phylogenetic relationships. However, plastid genome research studies in Campanulaceae have not been extensively performed. Only a few species (*Adenophora remotiflora*, *Campanula punctata*, *Campanula takesimana*, *Hanabusaya asiatica*, and *Trachelium caeruleum*) were sequenced for the whole cp genome [30, 35–38].

In this study, we report the chloroplast genome sequences of three *Adenophora* species (*A*. *divaricata*, *A*. *erecta*, *A*. *stricta*), and compared these sequences to five published Campanuloid chloroplast genomes. The specific goals of this study were to (1) confirm the genome features of the three *Adenophora* species, (2) the changing tendency of the chloroplast genome structure of Campanuloids, and (3) identify the divergence hotspot regions to provide information regarding useful molecular markers for future phylogenetic studies in *Adenophora*.

## Materials and methods

### Ethics statement

*Adenophora divaricata*, *A*. *erecta*, and *A*. *stricta* are not an endangered or protected species. We did not collect plant materials from any privately owned or protected area that required permission. The plant materials of *A*. *divaricata*, *A*. *erecta*, and *A*. *stricta* were collected at Mt. Hanseok (38°03'21"N, 128°16'52"E) in Gangwon-do Province, Ulleung Island (37°32'55"N, 130°54'19"E) in Gyeongsangbuk-do Province, and Sunyu Island (35°48'47"N, 126°24'31"E) in Jeollabuk-do Province in South Korea, respectively.

### Voucher specimens

The voucher specimen of three species was deposited at Kangwon National University Herbarium (KWNU). The voucher numbers are KWNU87503 (*A*. *divaricata*), KWNU80073 (*A*. *erecta*), and KWNU79662 (*A*. *stricta*).

### DNA extraction, sequencing, assembly and genome mapping

Total DNA was extracted from approximately 100 mg of fresh leaves using the DNeasy Plant Mini Kit (Qiagen Inc., Valencia, CA, USA). The total genomic DNA was used for sequencing by an Illumina MiSeq (Illumina Inc., San Diego, CA, USA) platform at LabGenomics (http://www.labgenomics.com/). Three *Adenophora* species were sequenced to produce 3,754,935–4,223,586 raw reads with a length of 301 bp. These paired-end reads were aligned of *Adenophora remotiflora* cp genome (accession no. KP889213) as a reference. After screening these paired-end reads through alignment with *A*. *remotiflora* cp genome, 325,221 to 560,114 reads were extracted. A *de novo* assembly was performed using Geneious v.7.1.8 (Biomatters Ltd., Auckland, New Zealand). The chloroplast genome coverage was estimated using the CLC Genomics Workbench v7.0.4 software (CLC-bio, Aarhus, Denmark). The cp genome coverages of the sequencing data of *A*. *divaricata*, *A*. *erecta*, and *A*. *stricta* were 524, 779, and 947×, respectively. In addition, the junction of large single copy (LSC), small single copy (SSC), and inverted repeat (IR) regions, along with the end points of inversion and IR contraction, were reconfirmed by PCR and Sanger sequencing.

The protein coding genes, tRNAs, and rRNAs in the plastid genome were predicted and annotated by Dual Organellar GenoMe Annotator (DOGMA) using the default parameters [39]. Based on this initial annotation, the putative starts and stops and the intron positions were determined by comparisons with homologous genes in other Campanulaceae cp genomes. tRNAs were confirmed using tRNAscan-SE [40]. A circular plastid genome map was drawn using the OGDRAW program [41].

### Comparative analysis of the genome structure and phylogenetic analysis

The chloroplast genome sequences of five Campanuloid species, i.e., *Adenophora remotiflora* (accession no. KP889213), *Campanula punctata* (accession no. KU198434), *Campanula takesimana* (accession no. KP006497), *Hanabusaya asiatica* (accession no. KJ477692), and *Trachelium caeruleum* (accession no. EU090187), were obtained from the GenBank. A genome structure comparison of eight Campanuloid species, including the three *Adenophora* species in this study and five downloaded Campanuloid species, was performed using Mauve [42].

In total, 76 protein coding genes (S1 Table) in the eight Campanuloid species and one outgroup (*Brighamia insignis*, KT372780) were compiled into a single file and aligned with MAFFT v.7 [43]. In addition, the *rpl23* and *infA* genes were excluded from the phylogenetic analysis data matrix, since most of these gene regions were deleted, and only a few regions existed. Before Maximum likelihood (ML) analysis, a search for the best fitting substitution model was performed using jModeltest v. 2.1.10 [44]. Based on the Akaike Infromation Criterion (AIC) and Akaike Information Criterion with Correction (AICc), GTR+I was the best model. ML analysis was performed using RAxML v7.4.2 [45] with 1,000 bootstrap replicates and the GTR+I model. Bayesian inference was performed using MrBayes v3.0b3 [46].

### Divergence hotspot identification in *Adenophora*

Four chloroplast genomes, including *A*. *divaricata*, *A*. *erecta*, *A*. *remotiflora* and *A*. *stricta*, were analyzed to identify rapidly evolving molecular markers that can be used in future phylogenetic studies in *Adenophora*. Both coding genes and non-coding region fragments >200 bp were extracted separately from each plastid genome by applying the “Extract” option in Geneious v.7.1.8 (Biomatters Ltd., Auckland, New Zealand). Then, the homologous loci were aligned individually using MAFFT v.7 [43]. To analyze the nucleotide diversity (Pi), the total number of mutations (Eta), the average number of nucleotide differences (K) and the parsimony informative characters (PICs) were determined using DnaSP v.5.10 [47].

## Results

### Genome features of the three *Adenophora* species

**Fig 1:**
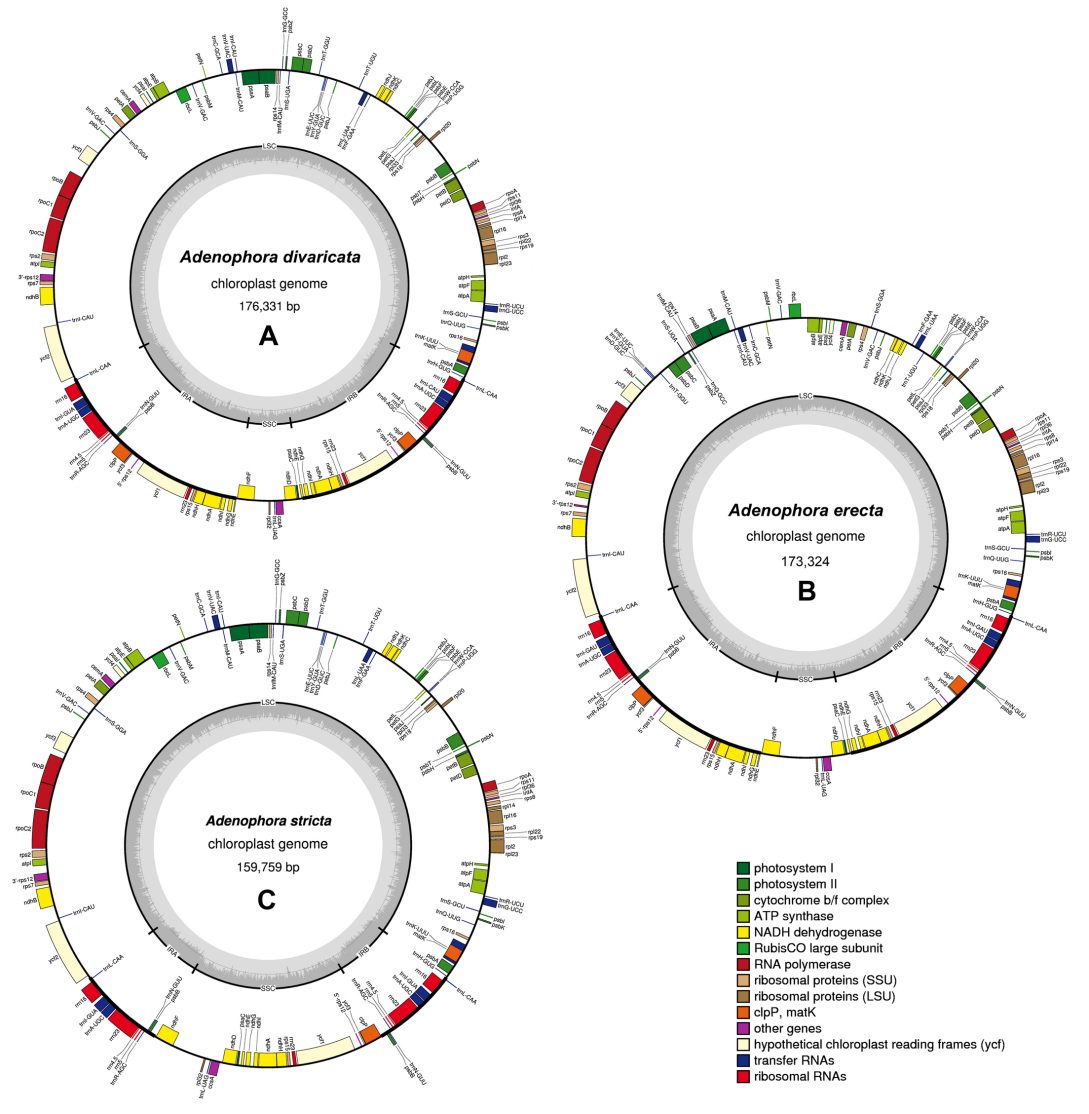
Chloroplast genomes of *A*. *divaricata*, *A*. *erecta*, and *A*. *stricta*. Genes inside the circle are transcribed clockwise, and genes outside the circle are transcribed counter-clockwise. The dark-gray inner circle corresponds to the GC content, and the light-gray represents the AT content. (A) *A*. *divaricata* chloroplast genome, (B) *A*. *erecta* chloroplast genome, and (C) *A*. *stricta* chloroplast genome.

**Fig 2:**
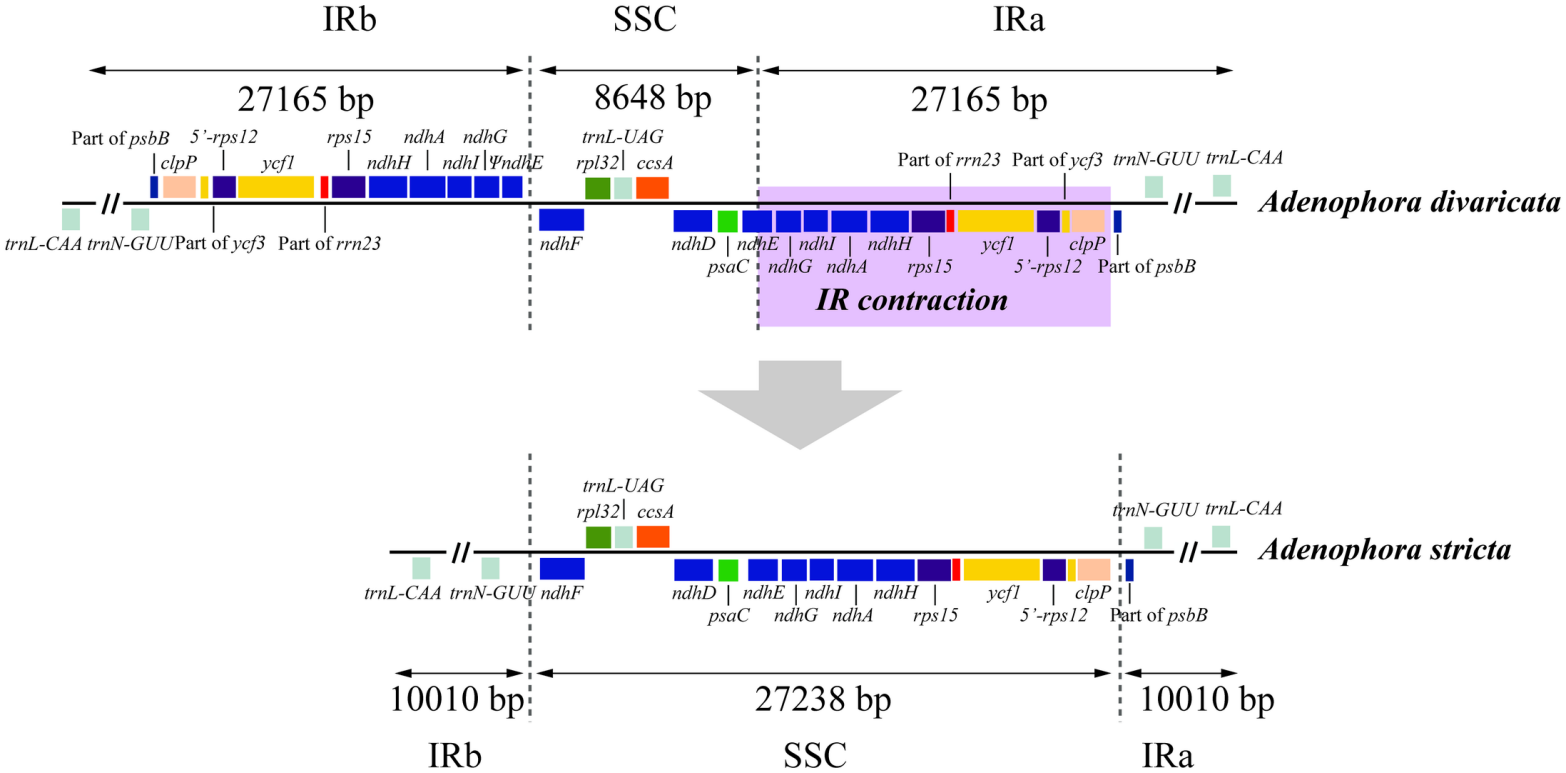
IR contraction in the *Adenophora stricta* chloroplast genome.

The chloroplast genomes of *A*. *divaricata* (accession no. KX462129), *A*. *erecta* (accession no. KX462130), and *A*. *stricta* (accession no. KX462131) have been submitted to GenBank of National Center for Biotechnology Information (NCBI). The plastid genome sizes of three *Adenophora* species ranged from 159,759 to 176,331 (Table 1 and Fig 1). The genome is composed of an LSC region (105,861–113,353 bp), SSC region (8,648–27,238 bp), and two IR copies (10,100–28,098 bp). Their overall GC contents are almost identical (38.5–38.7%). The chloroplast genomes of the three *Adenophora* species each contain 112 unique genes (Table 1). In the three *Adenophora* cp genomes, the following three genes (*rpl23*, *infA*, and *clpP*) are presumably nonfunctional: (1) only 46 bp of the 3’ end of *rpl23* exists, (2) only 202 bp in the middle of *infA* remain, and (3) only 38 bp in exon1 of *clpP* exists. Additionally, two tRNAs (*trnI-CAU* and *trnV-GAC*) and one gene (*psbJ*) had an additional one and two copies, respectively. In the chloroplast genomes of two species (*A*. *divaricata* and *A*. *erecta*), a portion of three genes (*psbB*, *ycf3*, and *rrn23*) was duplicated in the IRs. In *rps12*, the 3’ exon and 5’ exon were located in the LSC and IRs, respectively. However, the 5’ exon in *rps12*, and portion of *ycf3* and *rrn23* were located in the SSC in *A*. *stricta* due to the IR contraction (Fig 2).

**Table 1:** Comparison of chloroplast genome features of three *Adenophora* species.

Feature	*Adenophora divaricata*	*Adenophora erecta*	*Adenophora stricta*
Genome size (bp)	176,331	173,324	159,759
LSC	113,353	105,861	112,321
SSC	8,648	11,267	27,238
IR	27,165	28,098	10,100
Number of unique protein coding genes	78	78	78
Number of tRNAs	30	30	30
Number of rRNA	4	4	4
G+C (%)			
LSC	37.1	37.5	37.1
SSC	33.0	35.0	35.4
IR	42.2	41.8	51.0
Total genome	38.5	38.7	38.5

### Comparison of the chloroplast genome structure in eight Campanuloid species

The gene order changes in the plastid genomes of the eight Campanuloid species were confirmed only in the LSC region (except for *A*. *stricta*). The gene order among the three *Campanula s*. *str*. species (*C*. *punctata*, *C*. *takesimana*, and *T*. *caeruleum*) and between the two sect. *Remotiflorae* species (*A*. *erecta* and *A*. *remotiflora*) of *Adenophora* was exactly the same (S1 Fig).

**Fig 3:**
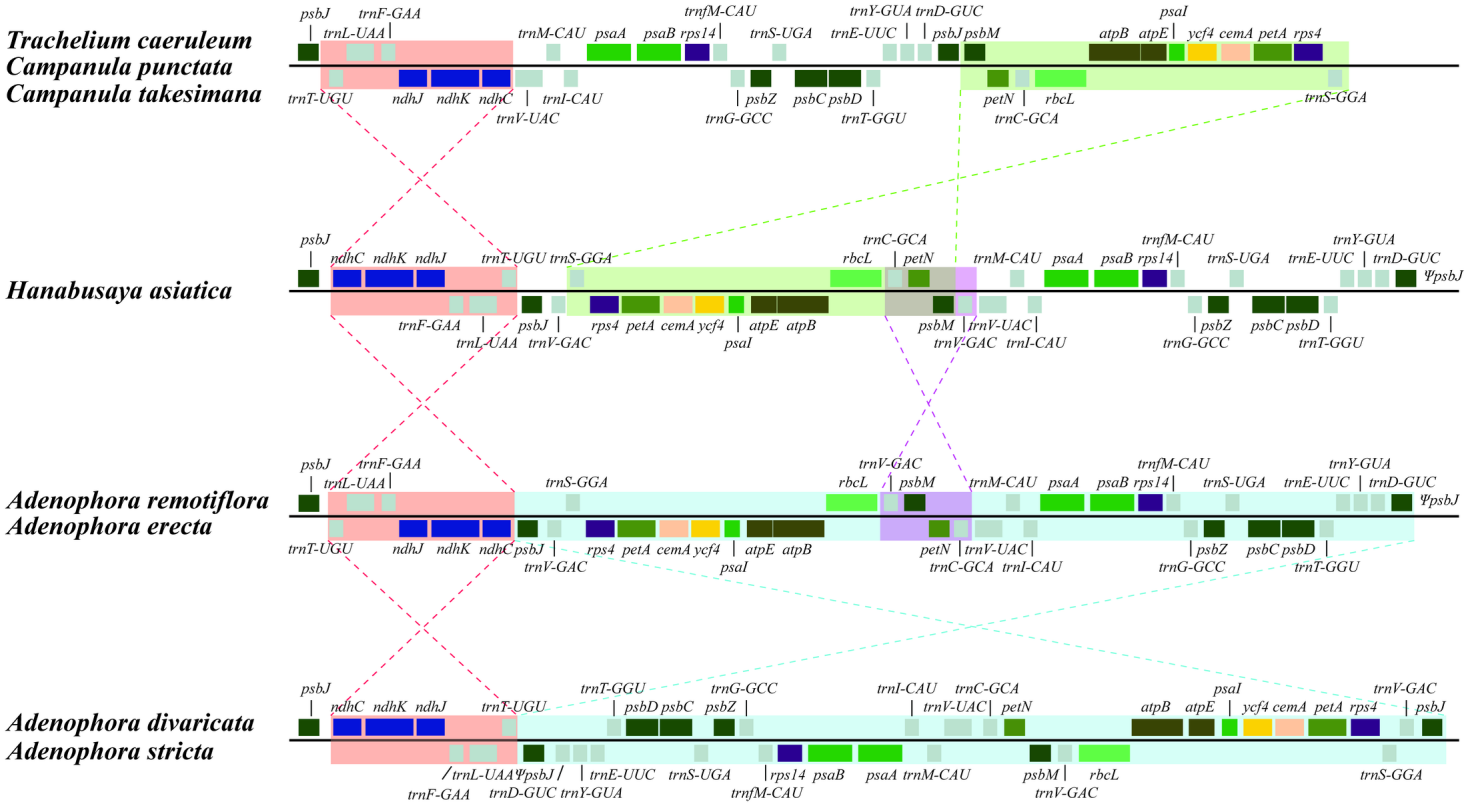
The gene order changes in the LSC regions in the eight Campanuloid chloroplast genomes.

Among the three *Campanula s*. *str*. species and *Hanabusaya*, two inversions of large gene blocks (*trnT-ndhC* and *psbM-trnS*) were found. In addition, two inversions between *Hanabusaya* and sect. *Remotiflorae* and between sect. *Remotiflorae* and the remaining *Adenophora* species (*A*. *divaricata* and *A*. *stricta*) were identified (Fig 3).

**Fig 4:**
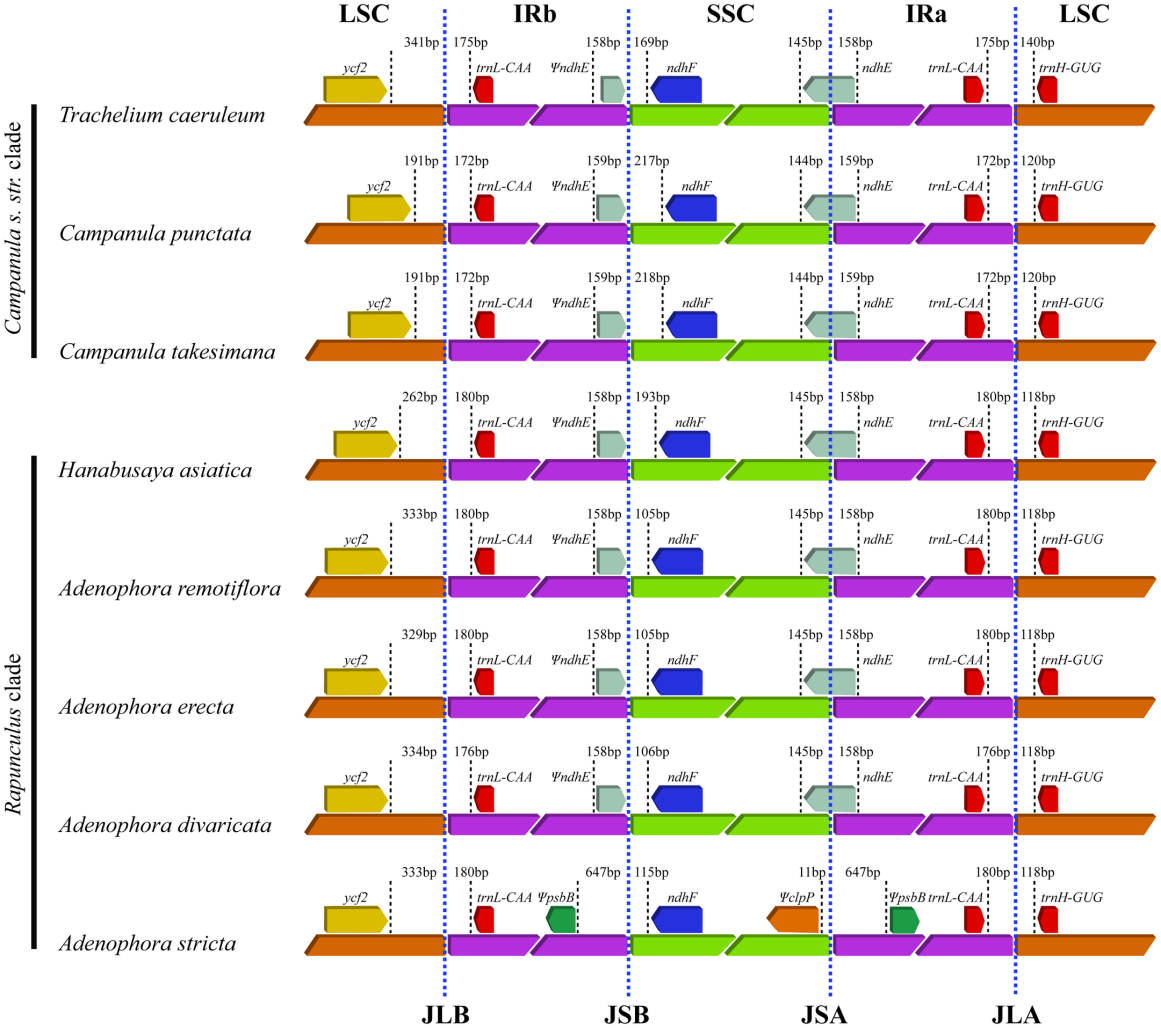
Comparison of the LSC, IR, and SSC junction positions in the eight Campanuloid chloroplast genomes.

The IR/LSC and IR/SSC borders in the eight Campanuloid plastid genomes were compared (Fig 4). Two genes (*ycf2* and *trnH-GUG*) in the eight Campanuloid species were all located in the LSC. *Ycf2* was separated from the LSC/IRb border by 191 bp (*C*. *punctata* and *C*. *takesimana*) to 341 bp (*T*. *caeruleum*), and *trnH-GUG* was separated from the IRa/LSC border by 118 bp (in five *Rapunculus* species) to 140 bp (*T*. *caeruleum*). In addition, *trnL-CAA* was found in the IR (separated from the LSC/IRb and IRa/LSC border by 172–180 bp), and *ndhF* was located in the SSC (separated from the IRb/SSC border by 105–218 bp). The IRb/SSC borders extended into *ndhE* to create a *ndhE* pseudogene in seven species (except for *A*. *stricta*). The lengths of the *ndhE* pseudogene were 159 bp in two species of *Campanula* and 158 bp in the remaining five species. In the *A*. *stricta* cp genome, the *psbB* pseudogene was duplicated in the IR near the IRb/SSC and SSC/IRa border, while the *clpP* pseudogene was located in the SSC adjacent to the SSC/IRa border.

Meanwhile, the cp genome of *A*. *stricta* lost duplication copies of *ndhG*, *ndhI*, *ndhA*, *ndhH*, *rps15*, *ycf1*, 5’ exon of *rps12*, and *clpP* due to the IR contraction (Fig 2).

### Phylogenetic analysis of eight Campanuloid species using 76 protein coding genes

The ML tree, which used 76 protein coding genes from the eight species, revealed that Campanuloids were monophyletic. The Campanuloids formed the following two clades: The *Campanula s*. *str*. clade and the *Rapunculus* clade. All nodes in the phylogenetic tree were strongly supported, with 100% bootstrap (BP) values and 1.00 Bayesian posterior probabilities (PP).

**Fig 5:**
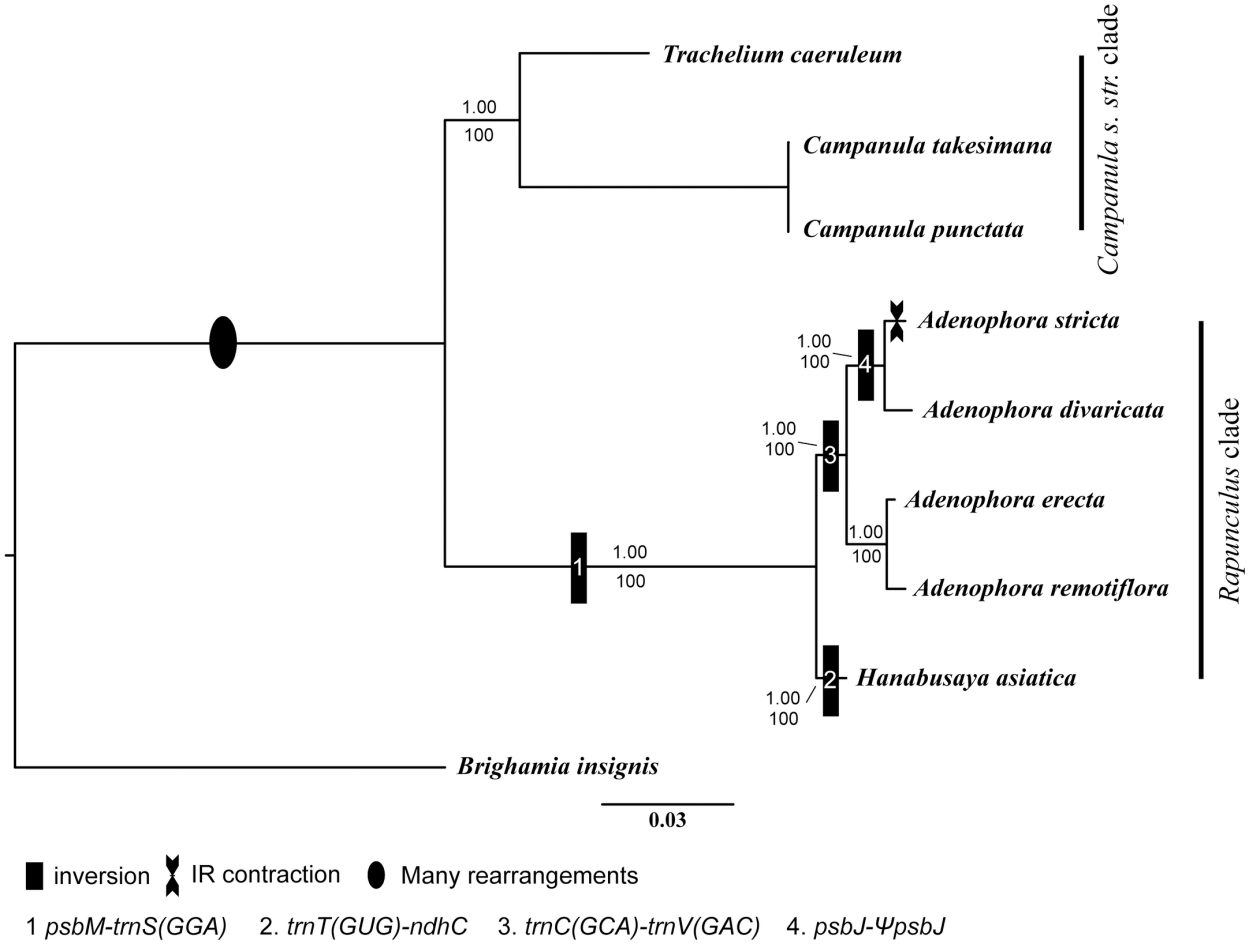
Phylogenetic tree reconstruction based on 76 protein coding genes using the ML. Bootstrap values are shown below the clades, and Bayesian posterior probabilities are shown above the clades.

In the *Campanula s*. *str*. clade, *T*. *caeruleum* is the earliest diverging lineage, which was identified as a sister to the other species in this group. Within the *Rapunculus* clade, *H*. *asiatica* formed a basal branch and was a sister to all other taxa. Additionally, *A*. *erecta* and *A*. *remotiflora* as well as *A*. *divaricata* and *A*. *stricta* formed a clade (Fig 5).

### Divergence sequence hotspot regions in *Adenophora*

**Fig 6:**
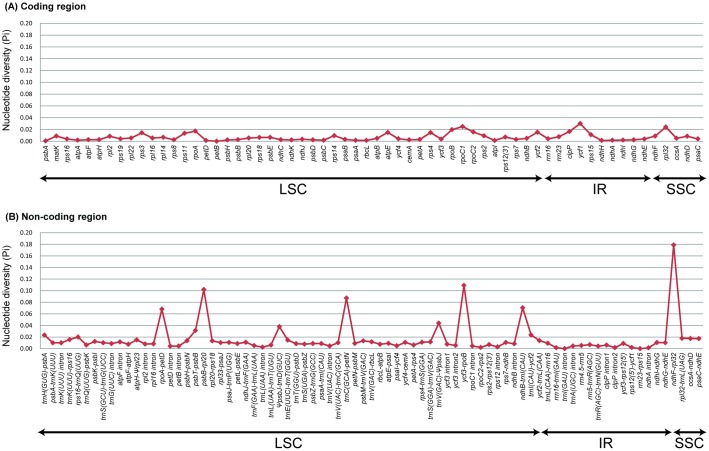
Comparison of the Pi value in four *Adenophora* species. (A) Coding genes and (B) Non-coding regions.

In total, 141 loci (62 coding genes and 79 non-coding regions) were compared across the four *Adenophora* species. The Pi values ranged from 0 (*petB*, *trnI* intron, and *rrn23*-*rps15*) to 0.17886 (*ndhF*-*rpl32*). Ten of these loci, i.e., *rpoA*-*petD* (0.06819), *psbT*-*psbB* (0.03145), *psbB*-*rpl20* (0.10200), *ΨpsbJ*-*trnD* (0.03801), *trnC*-*petN* (0.08722), *trnV*-*ΨpsbJ* (0.04408), *ycf3*-*rpoB* (0.10918), *ndhB*-*trnI* (0.07050), *ndhF*-*rpl32* (0.17886), and *ycf1* (0.03011), showed high values (greater than 0.03). Additionally, eight of these regions were located in the LSC, while *ycf1* and *ndhF*-*rpl32* were located in the IR and SSC, respectively (Fig 6 and S2 Table).

## Discussion

### Chloroplast genome organization in Campanuloids

The LSC, SSC, and IR regions in the three *Adenophora* cp genomes varied in length. In particular, the length of the IR regions in *A*. *stricta* was significantly shorter than that in the other species, which is thought to be due to the IR contraction. The length of the LSC region in *A*. *erecta* was approximately 7,000 bp shorter than that in the other species, and the lengths of the SSC and IR regions in *A*. *erecta* were approximately 2,500 bp and 900 bp longer than those in the *A*. *divaricata* cp genome, respectively. It is believed that the length difference in the LSC region was due to a change in the length of the intergenic spacer (IGS) in *psbJ-ndhC*, *trnT(UGU)-psbJ* and *psbJ-ycf3*, which is the end point of the inversion. Additionally, the length difference in the SSC and IR regions was identified to be caused by the length difference in the IGS regions in portion of *ycf3-rps12(5’)*, which is located in the IR regions, and *ndhF-rpl32*, which is located in the SSC region, regardless of the inversion.

The Campanuloid plastomes were identified to have a very different gene order when compared to the cp genome of *B*. *insignis* belonging to Campanulaceae *s*. *l*. This is important evidence that many rearrangements in the cp genome occurred among the Campanulaceae species. However, it is very difficult to estimate the exact gene order change process by only the cp genomes analyzed so far, since the plastid genome of species belonging to Wahlenbergioids and Platycodonoids has not been analyzed in any species to date.

The results of this study revealed that the chloroplast genome structure of two species of *Campanula* (*C*. *punctata* and *C*. *takesimana*) was almost identical to that of *Trachelium* regarding the gene order and duplication of certain genes. In addition, we confirmed that the genome structures two *Remotiflorae* species (*A*. *erecta* and *A*. *remotiflora*) of *Adenophora* were the same. Therefore, we believe that the cpDNAs of members of *Campanula s*. *str*. and sect. *Remotiflorae* evolved via identical routes. Genome structure modifications in the phylogenetic groups of *Campanula s*. *str*. and *Rapunculus* were identified only in the LSC region (except for *A*. *stricta*). Duplicated copies of *psbJ* and *trnV-GAC* were located at the inversion endpoint (Fig 3). This duplication might be attributed to inversions, and these duplicated genes might have caused multiple inversion events within Campanuloids.

Many interspecific and intergeneric relationships of Campanuloids currently remain unclear. Specifically, the phylogenetic relationships among *Adenophora* are currently unresolved. In this study, the genome structures of each section of *Adenophora*, such as sect. *Remotiflorae* (*A*. *erecta* and *A*. *remotiflora*), sect. *Platyphyllae* (*A*. *divaricata*), and sect. *Microdiscus* (*A*. *stricta*) were different. Therefore, we believe that the chloroplast genome structure can be used as an important characteristic to differentiate the *Adenophora*.

Among the 11 plastid-encoded *ndh* genes, a loss of function of *ndhK* in the chloroplast genome has been reported in *Pinus thunbergii* and *Phalaenopsis aphrodite* [48–49]. Haberle et al. [30] have suggested that *ndhK* might be a pseudogene in *T*. *caeruleum* because it contains multiple internal stop codons. We compared *ndhK* in *T*. *caeruleum* to that in the seven Campanuloid species. The results of the comparison revealed that *ndhK* in *T*. *caeruleum* was not a pseudogene. Its earlier identification as a pseudogene might be due to an error in the annotation process. In conclusion, *ndhK* in the chloroplast genomes of Campanuloid species is a functional gene (S2 Fig).

### Phylogenetic implications

Recent phylogenetic studies [4, 6–7] have proposed that Campanuloids can be divided into the following two clades: *Campanula s*. *str*. clade and *Rapunculus* clade. The results of this study were based on 76 protein coding genes, and these two clades were well supported and clearly distinguished by the gene order in the plastid genome structure (Fig 5).

The phylogenetic tree constructed in this study showed that *A*. *erecta* and *A*. *remotiflora* (sect. *Remotiflorae*) formed a sister clade to the *A*. *divaricata* (sect. *Platyphyllae*) and *A*. *stricta* (sect. *Microdiscus*) clade (Fig 5). Therefore, it is hypothesized that sect. *Remotiflora* had the earliest divergence among the section of *Adenophora*. Additionally, sect. *Platyphyllae* had an earlier divergence from a common ancestor than sect. *Microdiscus* because *A*. *stricta* had a unique IR structure among the Campanulaceae due to the IR contraction (Figs 2 and 5).

Meanwhile, *H*. *asiatica* is a very important plant resource since it is monotypic and one of the six endemic genera in Korea. It was first described as *Symphyandra asiatica* Nakai [50] due to its connate anthers but was segregated into a new genus, *Hanabusaya*, based on its morphological characteristics [51]. However, the phylogenetic position of *Hanabusaya* as an endemic genus remain unclear despite the many studies that have been performed [7, 18, 24–26, 52]. In this study, *Hanabusaya* formed a sister clade to the *Adenophora* clade. Thus, *Hanabusaya* has evolved through a different evolutionary route than the morphologically similar *Campanula s*. *str*. species, including *Campanula* and *Symphyandra*. Additionally, *Hanabusaya* has a unique cp genome structure compared to that in the other species used in this study (Fig 3). Therefore, the phylogenetic position of *Hanabusaya* was well supported in this study as an endemic genus. However, only a few species were included in this study, and thus, we believe that further studies that include various species are needed to clarify the phylogenetic position of *Hanabusaya*.

### Useful molecular markers information for the phylogeny of *Adenophora*

Despite the many phylogenetic studies performed [4, 6, 25–26], the phylogenetic relationships among the *Adenophora* species remain unclear. We believe that this is due to nucleotide variations in molecular markers, such as *atpB*, *matK*, *rbcL*, *atpB*-*rbcL*, *atpF*-*atpH*, *rpl16*, *rpoC1*, and *trnL*-*trnF*, which were very low in previous studies (Fig 6 and S2 Table).

The results of this study showed that the nucleotide diversity in ten regions, including one coding gene and nine non-coding regions, had relatively high calculated values (>0.03). However, among these regions, one non-coding region (*ycf3*-*rpoB*) was not considered suitable as a phylogenetic marker because it had a very small number of PICs compared to the Eta and/or aligned length (S2 Table).

Therefore, nine regions (*rpoA*-*petD*, *psbT*-*psbB*, *psbB*-*rpl20*, *ΨpsbJ*-*trnD*, *trnC*-*petN*, *trnV*-*ΨpsbJ*, *ndhB*-*trnI*, *ndhF*-*rpl32*, and *ycf1*) are considered useful markers for evaluating the phylogenetic relationships among the *Adenophora* species.

## Conclusions

We provide the first report of the complete plastid genome sequences of *A*. *divaricata*, *A*. *erecta*, and *A*. *stricta* and compared these sequences to those of five Campanuloid species in Campanulaceae. The results of the genome structure comparison confirmed many inversions. The phylogenetic analyses showed that Campanuloids were divided two major groups. The divergence hotspots clarified in this study could be used as molecular markers that will be helpful for elucidating the phylogenetic relationships among the *Adenophora* species.

## Supporting information

S1 Fig The gene order comparison among eight Campanuloid chloroplast genomes using MAUVE program. (TIF)

S2 Fig Nucleotide alignment of chloroplast copies of *ndhK* genes for eight Campanuloid species. (TIF)

S1 Table The length and aligned length of 76 protein coding genes used for phylogenetic analyses. (XLSX)

S2 Table Eta, Pi value, and PICs of 141 homologous loci. (XLSX)

## References

[1] Shetler SG, Morin NR. Seed morphology in North American Campanulaceae. Annals of the Missouri Botanical Garden. 1986; 73:653–688.

[2] Kolakovskii AA. The conspectus of the system of the old world Campanulaceae. Botanicheskii Zhurnal. 1994; 79:109–124.

[3] Takhtajan A. Diversity and Classification of Flowering Plants. New York: Columbia University Press; 1997 p. 405–412.

[4] Haberle RC, Dang A, Lee T, Penaflor C, Cortes-Burns H, Oestreich A, et al Taxonomic and biogeographic implications of a phylogenetic analysis of the Campanulaceae based on three chloroplast genes. Taxon. 2009; 58:715–734.

[5] Schönland S. Campanulaceae In: Engler A, Prantl K, editors. Die natürlichen Planzenfamilien. Leipzig: Wilhelm Engelmann; 1889 p. 40–70.

[6] Eddie WMM, Shulkina T, Gaskin J, Haberle RC, Jansen RK. Phylogeny of Campanulaceae *s*. *str*. inferred from ITS sequences of nuclear ribosomal DNA. Annals of the Missouri Botanical Garden. 2003; 90:554–575.

[7] Cheon KS, Yoo KO. Phylogeny of *Hanabusaya* (Campanulaceae), a Korean endemic, based on ITS sequences of nuclear ribosomal DNA. Journal of Systematics and Evolution. 2013; 51:704–714. doi: 10.1111/jse.12039

[8] Fischer FEL. Adumbratio generis Adenophorae. Mémories de la Société Impériale des Naturalistes de Moscou. 1823; 6:165–169.

[9] Korshinsky S. Untersuchungen über die Russischen Adenophora-Arten Mémories de ľAcadémie Impériale des Sciences de St. Pétersbourg. 42:1–41.

[10] Fedorov AA. Flora of the U.S.S.R vol 24: Dipsacaceae, Cucurbitaceae, Campanulaceae. Moskva-Leningrad: Izdatel’stvo Akademii Nauk SSSR; 1957 pp. 246–272.

[11] Baranov AI. Materials to the monograph of the species of *Adenophora* of N.E.China. Quarterly Journal of the Taiwan Museum. 1963; 16:143–179.

[12] Hong DY. *Adenophora* Fisch In: Hong DY, Lian YS, Shen LD, editors. Flora reipublicae popularis sinicae. vol 73(2). Beijing: Science Press; 1983 pp. 92–139.

[13] Fu CX, Liu MY. Study on the taxonomy of *Adenophora* Fischer in Heilongjiang Province. Journal of Harbin Normal University (Natural Science). 1986; 2:41–52.

[14] Okazaki J. *Adenophora* Fisch In: Iwatsuki K, Yamazaki T, Boufford DE, Ohba H, editors. Flora of Japan. 3a. Tokyo: Kodansha Ltd; 1993 pp. 406–410.

[15] Lee JK. A taxonomic study of the genus Adenophora in Korea. M.Sc. Thesis, Sungkyunkwan Universiy. 1989.

[16] Yoo KO. Taxonomic studies on the Korean Campanulaceae. Ph.D. Thesis, Kangwon National University. 1995.

[17] Tu PE, Chen HB, Xu GJ, Xu LS. Classification and evolution of the genus *Adenophora* Fischer in China. Acta Botanica Boreali-Occidentalia Sinica. 1998; 18:613–621.

[18] Lee ST, An YM, Park KR. Palynological relationship of *Hanabusaya asiatica* Nakai within the Campanulaceae. Korean Journal of Plant Taxonomy. 1986; 16:25–37.

[19] Yoo KO, Lee WT, Lim HT. Comparative studies on the *Hanabusaya asiatica* and its allied groups. 1. External morphology and anatomical characters. Korean Journal of Plant Resources. 1995; 8:223–236.

[20] Yoo KO, Lee WT, Lee JY, Lim HT. Comparative studies on the *Hanabusaya asiatica* and its allied groups. 2. Ultrastructure of epidermis, palynological characters and isozyme pattern. Korean Journal of Plant Resources. 1995; 8:303–318.

[21] Yoo KO, Lee WT, Kim NS, Kim JH, Lim HT. Comparative studies on the *Hanabusaya asiatica* and its allied groups based on randomly amplified polymorphic DNA (RAPD) analysis. Horticulture Environment and Biotechnology. 1996; 37:324–328.

[22] Tu P, Niu Y, Xu L, Xu G. Microscopic identification of the powder of roots of genus *Adenophora*. I. The roots of sect. *Basiphyllae* and sect. *Pachydiscus*. Zhongguo Zhong Yao Za Zhi. 1996; 21:581–585. 9772625

[23] Tu P, Niu Y, Xu L, Xu G. Microscopic identification of the powder of roots of genus *Adenophora*: II. The roots of sect. *Remotiflorae* and sect. *Adenophora*. Zhongguo Zhong Yao Za Zhi. 1997; 22:67–72. 10743193

[24] Kim YD, Lee JK, Suh YB, Lee ST, Kim SH, Jansen RK. Molecular evidence for the phylogenetic position of *Hanabusaya asiatica* Nakai (Campanulaceae), an endemic species in Korea. Journal of Plant Biology. 1999; 42:168–173.

[25] Kim KA, Yoo KO. Phylogenetic relationships of Korean Campanulaceae based on PCR-RFLP and ITS sequences. Korean Journal of Plant Taxonomy. 2011; 41:119–129.

[26] Kim KA, Yoo KO. Phylogenetic relationships of Korean Campanulaceae based on chloroplast DNA sequences. Korean Journal of Plant Taxonomy. 2012; 42:282–293.

[27] Crowl AA, Mavrodiev E, Mansion G, Haberle R, Pistarino A, Kamari G, et al Phylogeny of Campanuloideae (Campanulaceae) with emphasis on the utility of nuclear pentatricopeptide repeat (PPR) genes. PLOS one. 2014; 9(4):e94199 doi: 10.1371/journal.pone.0094199 24718519 10.1371/journal.pone.0094199 PMC3981779

[28] Lee S, Lee J, Kim S. A new species of *Adenophora* (Campanulaceae) from Korea. Journal of Plant Research. 1997; 110:77–80. doi: 10.1007/BF02506845 27520046 10.1007/BF02506845

[29] Kim KA. Phylogenetic study of the genus Adenophora (Campanulaceae). Ph.D. Thesis, Kangwon National University. 2016.

[30] Haberle RC, Fourcade HM, Boore JL, Jansen RK. Extensive rearrangements in the chloroplast genome of *Trachelium caeruleum* are associated with repeats and tRNA genes. Journal of Molecular Evolution. 2008; 66:350–361. doi: 10.1007/s00239-008-9086-4 18330485 10.1007/s00239-008-9086-4

[31] Cosner ME, Raubeson LA, Jansen RK. Chloroplast DNA rearrangements in Campanulaceae: phylogenetic utility of highly rearranged genomes. BMC Evolutionary Biology. 2004; 4:27 doi: 10.1186/1471-2148-4-27 15324459 10.1186/1471-2148-4-27 PMC516026

[32] Kim YK, Park CW, Kim KJ. Complete chloroplast DNA sequences from a Korean endemic genus, *Megaleranthis saniculifolia* and its evolutionary implications. Molecules and Cells. 2009; 27:365–381. doi: 10.1007/s10059-009-0047-6 19326085 10.1007/s10059-009-0047-6

[33] Wicke S, Schneeweiss GM, de Pamphilis CW, Muller KF, Quandt D. The evolution of the plastid chromosome in land plants: gene content, gene order, gene function. Plant Molecular Biology. 2011; 76:273–297. doi: 10.1007/s11103-011-9762-4 21424877 10.1007/s11103-011-9762-4 PMC3104136

[34] Cosner ME, Jansen RK, Palmer JD, Downie SE. The highly rearranged chloroplast genome of *Trachelium caeruleum* (Campanulaceae): multiple inversions, inverted repeat expansion and contraction, transposition, insertions/deletions, and several repeat families. Current Genetics. 1997; 31:419–429. 9162114 10.1007/s002940050225

[35] Cheon KS, Yoo KO. Complete chloroplast genome sequence of *Hanabusaya asiatica* (Campanulaceae), an endemic genus to Korea. Mitochondrial DNA Part A. 2016; 27:1629–1631. doi: 10.3109/19401736.2014.958702 25208164 10.3109/19401736.2014.958702

[36] Cheon KS, Kim KA, Jang SK, Yoo KO. Complete chloroplast genome sequence of *Campanula takesimana* (Campanulaceae), an endemic to Korea. Mitochondrial DNA Part A. 2016; 27:2169–2171. doi: 10.3109/19401736.2014.982610 25423504 10.3109/19401736.2014.982610

[37] Kim KA, Cheon KS, Jang SK, Yoo KO. Complete chloroplast genome sequence of *Adenophora remotiflora* (Campanulaceae). Mitochondrial DNA Part A. 2016; 27:2963–2964. doi: 10.3109/19401736.2015.1060461 26119125 10.3109/19401736.2015.1060461

[38] Yoo KO, Cheon KS, Kim KA. Complete chloroplast genome sequence of *Campanula punctata* Lam. (Campanulaceae). Mitochondrial DNA Part B. 2016; 1:192–193. doi: 10.1080/23802359.2016.1149791 10.1080/23802359.2016.1149791 PMC7871831 33644338

[39] Wyman SK, Jansen RK, Boore JL. Automatic annotation of organellar genomes with DOGMA. Bioinformatics. 2004; 20:3252–3255. doi: 10.1093/bioinformatics/bth352 15180927 10.1093/bioinformatics/bth352

[40] Schattner P, Brooks AN, Lowe TM. The tRNAscan-SE, snoscan and snoGPS web servers for the detection of tRNAs and snoRNAs. Nucleic Acids Research. 2005; 33:W686–W689. doi: 10.1093/nar/gki366 15980563 10.1093/nar/gki366 PMC1160127

[41] Lohse M, Dechsel O, Bock R. OrganellarGenomeDRAW (OGDRAW): a tool for the easy generation of high-quality custom graphical maps of plastid and mitochondrial genomes. Current Genetics. 2007; 52:267–274. doi: 10.1007/s00294-007-0161-y 17957369 10.1007/s00294-007-0161-y

[42] Darling ACE, Mau B, Blattner FR, Perna NT. Mauve: Multiple alignment of conserved genomic sequence with rearrangements. Genome Research. 2004; 14:1394–1403. doi: 10.1101/gr.2289704 15231754 10.1101/gr.2289704 PMC442156

[43] Katoh K, Misawa K, Kuma K, Miyata T. MAFFT: a novel method for rapid multiple sequence alignment based on fast Fourier transform. Nucleic Acids Research. 2002; 30:3059–3066. doi: 10.1093/nar/gkf436 12136088 10.1093/nar/gkf436 PMC135756

[44] Darriba D, Taboada GL, Doallo R, Posada D. jModelTest 2: more models, new heuristics and parallel computing. Nature Methods. 2012; 9(8):772 doi: 10.1038/nmeth.2109 22847109 10.1038/nmeth.2109 PMC4594756

[45] Stamatakis A. RAxML-VI-HPC: maximum likelihood-based phylogenetic analyses with thousands of taxa and mixed models. Bioinformatics. 2006; 22:2688–2690. doi: 10.1093/bioinformatics/btl446 16928733 10.1093/bioinformatics/btl446

[46] Huelsenbeck JP, Ronquist R. MrBayes: Bayesian inference of phylogeny. Bioinformatics. 2001; 17:754–755. 11524383 10.1093/bioinformatics/17.8.754

[47] Librado P, Rozas J. DnaSP v5: software for comprehensive analysis of DNA polymorphism data. Bioinformatics. 2009; 25:1451–1452. doi: 10.1093/bioinformatics/btp187 19346325 10.1093/bioinformatics/btp187

[48] Wakasugi T, Tsudzeki J, Ito S, Nakashima K, Tsudzuki T, Sugiura M. Loss of all *ndh* genes as determined by sequencing the entire chloroplast genome of the black pine *Pinus thunbergii*. PNAS. 1994; 91:9794–9798. 7937893 10.1073/pnas.91.21.9794 PMC44903

[49] Chang CC, Lin HC, Lin IP, Chow TY, Chen HH, Chen WH, et al The chloroplast genome of *Phalaenopsis aphrodite* (Orchidaceae): comparative analysis of evolutionary rate with that of Grasses and its phylogenetic implications. Molecular Biology and Evolution. 2006; 23:279–291. doi: 10.1093/molbev/msj029 16207935 10.1093/molbev/msj029

[50] Nakai T. Plantae novae Asiaticae. Botanical Magazine, Tokyo. 1909; 23:185–192.

[51] Nakai T. Flora Koreana II. Journal of the College of Science, Imperial University of Tokyo. 1911; 31:64–68.

[52] Park KR, Ko MS. Taxonomic position of *Hanabusaya asiatica* Nakai within Korean Campanulaceae: phylogenetic analysis using morphological data. Journal of Basic Science Research Institute, Kyungnam University. 2000; 14:171–181.

