# Supplementary figures and images for: The complete chloroplast genome sequences of three Adenophora species and comparative analysis with Campanuloid species (Campanulaceae)
Source: PLoS One. 2017 Aug 22;12(8):e0183652. doi: 10.1371/journal.pone.0183652 (PMC5568750; doi:10.1371/journal.pone.0183652)

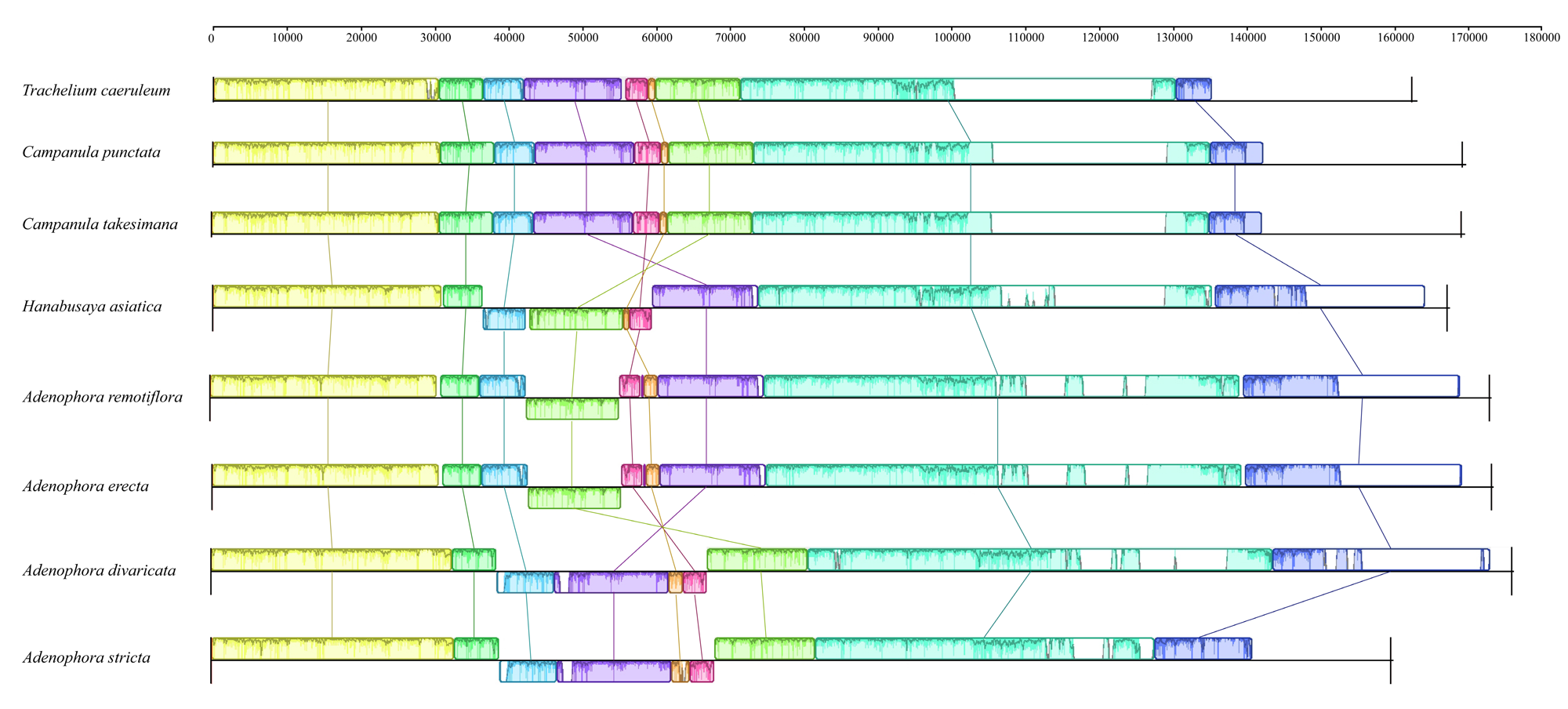

Supplement: S1 Fig — (TIF) [file pone.0183652.s001.tif]

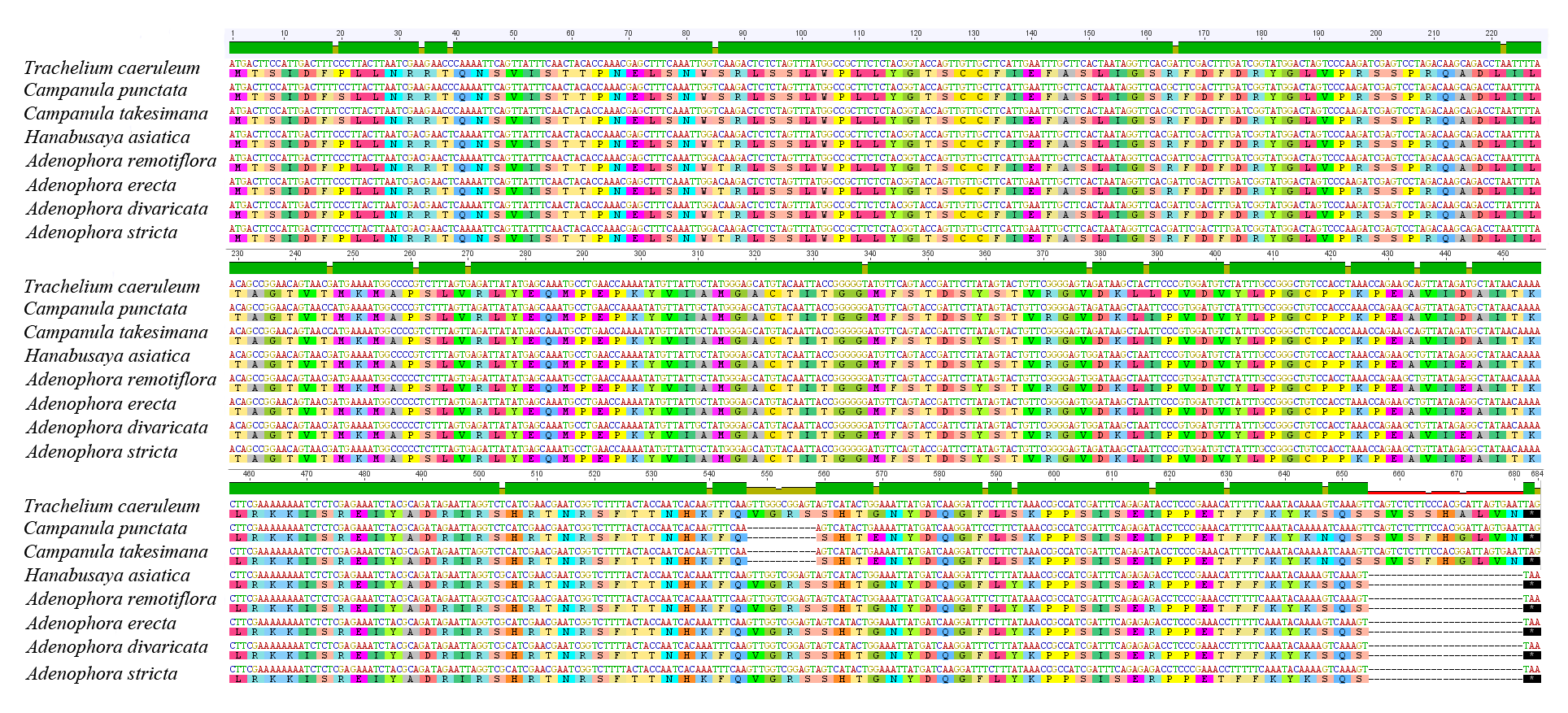

Supplement: S2 Fig — (TIF) [file pone.0183652.s002.tif]
